# Interleukin-19: A Constituent of the Regulome That Controls Antigen Presenting Cells in the Lungs and Airway Responses to Microbial Products

**DOI:** 10.1371/journal.pone.0027629

**Published:** 2011-11-15

**Authors:** Carol Hoffman, Sung-Hyun Park, Eleen Daley, Claire Emson, Jennifer Louten, Maureen Sisco, Rene de Waal Malefyt, Gabriele Grunig

**Affiliations:** 1 Department of Environmental Medicine, New York University School of Medicine, Tuxedo, New York, United States of America; 2 Department of Pathology, St. Luke's Roosevelt Hospital, New York, New York, United States of America; 3 Merck Research Laboratories (formerly Schering Plough Biopharma), Palo Alto, California, United States of America; 4 Division of Pulmonary Medicine, Department of Medicine, New York University School of Medicine, New York, New York, United States of America; University of Southern California, United States of America

## Abstract

**Background:**

Interleukin (IL)-19 has been reported to enhance chronic inflammatory diseases such as asthma but the *in vivo* mechanism is incompletely understood. Because IL-19 is produced by and regulates cells of the monocyte lineage, our studies focused on *in vivo* responses of CD11c positive (CD11c+) alveolar macrophages and lung dendritic cells.

**Methodology/Principal Findings:**

IL-19-deficient (IL-19-/-) mice were studied at baseline (naïve) and following intranasal challenge with microbial products, or recombinant cytokines. Naïve IL-19-/- mixed background mice had a decreased percentage of CD11c+ cells in the bronchoalveolar-lavage (BAL) due to the deficiency in IL-19 and a trait inherited from the 129-mouse strain. BAL CD11c+ cells from fully backcrossed IL-19-/- BALB/c or C57BL/6 mice expressed significantly less Major Histocompatibility Complex class II (MHCII) in response to intranasal administration of lipopolysaccharide, Aspergillus antigen, or IL-13, a pro-allergic cytokine. Neurogenic-locus-notch-homolog-protein-2 (Notch2) expression by lung monocytes, the precursors of BAL CD11c+ cells, was dysregulated: extracellular Notch2 was significantly decreased, transmembrane/intracellular Notch2 was significantly increased in IL-19-/- mice relative to wild type. Instillation of recombinant IL-19 increased extracellular Notch2 expression and dendritic cells cultured from bone marrow cells in the presence of IL-19 showed upregulated extracellular Notch2. The CD205 positive subset among the CD11c+ cells was 3-5-fold decreased in the airways and lungs of naïve IL-19-/- mice relative to wild type. Airway inflammation and histological changes in the lungs were ameliorated in IL-19-/- mice challenged with Aspergillus antigen that induces T lymphocyte-dependent allergic inflammation but not in IL-19-/- mice challenged with lipopolysaccharide or IL-13.

**Conclusions/Significance:**

Because MHCII is the molecular platform that displays peptides to T lymphocytes and Notch2 determines cell fate decisions, our studies suggest that endogenous IL-19 is a constituent of the regulome that controls both processes *in vivo*.

## Introduction

IL-19 is a member of the IL-10 family of cytokines whose biological role has remained incompletely understood. IL-19 is thought to be significant for human health because increased IL-19 levels have been reported in asthma [1], [2], [3], [4]. Furthermore, IL-19 has also been associated with psoriasis, an autoimmune disease of the skin [5], [6], [7], [8], [9], [10], [11], [12], and rheumatoid arthritis [13]. The unique effects for human health by the structurally closely related IL-19, IL-20, and IL-24 [14], that all signal via the transcription factor Signal Transducer and Activator of Transcription 3 (STAT3) [15], [16], [17], [18], are thought to be mediated by distinct utilization and expression of receptors: IL-20 receptor A/B (IL-20RA/B) for signaling by IL-19, 20, 24; and IL-22R/IL-20RB for signaling by IL-20 and IL-24 [18]. IL-20RB is the most widely expressed of the IL-19 receptor subunits. In lungs, epithelial cells and infiltrating immune cells have been reported to express both IL-20RA and IL-20RB [18]. Hematopoietic progenitor cells and non-hematopoietic cells like epithelial cells and fibroblasts express IL-20RA/B, while mature T cells and B cells express the IL-20RA/B at low levels or not at all [19].

The strongest evidence for the biological significance of IL-19 thus far has been provided by studies indicating that the human *il19* gene (but not the *il20* or *il24* genes) and the gene for one of its receptor components, the *il20RB,* has been under evolutionary pressure from human pathogens (helminth parasites, bacteria and viruses) [20]. The same study [20] additionally found evidence of evolutionary pressure on the human *il20RA* gene by parasites. Because the immune response has evolved for protection from infectious diseases, these findings suggest the significance of IL-19 and both of its receptors for human health.

The immune defense from helminth parasites requires intact T helper 2 (Th2) responses and the elaboration of Th2 mediators, such as IL-13 [21], [22]. These same immune responses and immune mediators can produce pathology, most prominently asthma [23], [24], [25]. Therefore, both the suggested evolutionary pressure by parasites on the human *il19*, *IL20RB* and *IL20RA* genes [20] and the association of IL-19 with asthma [1], [2], [3], [4] imply a critical role of IL-19 for the control of Th2 responses. To address the gap in our knowledge of the *in vivo* role of IL-19 in responses of the lungs to inflammatory stimuli, the present study was designed to determine the phenotype of IL-19-/- mice on three background-strains: 129xBL6, C57BL/6, and BALB/c. Our data indicate that IL-19 is a constituent of the regulome that controls responses of CD11c+ cells and monocytes in the lungs *in vivo*, in particular cell surface expression of MHCII, CD205 and Notch2.

## Results

### Endogenous IL-19 affects the cellular composition of bronchoalveolar lavage (BAL) fluid in 129xBL6 mice

**Figure 1:**
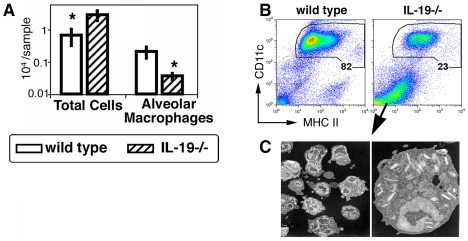
Decreased abundance of CD11c+ cells in the BAL of naïve IL-19-/- 129xBL6 mice. (**A**) Bar graphs (white – wild-type, striped – IL-19-/-) show means and SEM of numbers of total cells and CD11c+ cells in the BAL. One representative independent experiment of 5 is shown (n = 4-5 per group per study), *p<0.05, Mann-Whitney-U test. (**B**) Dot plots show CD11c versus MHCII staining of BAL cells. The percentages of the gated CD11c+ cells are indicated. One representative of 20 independent experiments is shown. (**C**) Electron micrographs of marker negative cells in IL-19-/- mice. The marker negative cells were purified by cell sorting from a pool of BAL samples from groups of 20 to 25 mice of each genotype: wild type or IL-19-/-. Note that cells from the BAL of IL-19KO mice were eosinophils. Only debris was harvested from the BAL of wild type mice (not shown).

Naive IL-19-/- 129xBL6 mice had significantly decreased CD11c+ cells in the BAL (Fig. 1A). Although the total cell number in the BAL in IL-19-/- 129xBL6 mice was significantly increased relative to wild-type mice (Fig. 1A), it was variable and overlapped with wild type. A decrease in the abundance of CD11c+ cells in the airways is typically seen in airway inflammation, induced for example with lipopolysaccharide, antigen, recombinant IL-13 or Interferon gamma. In all these cases of inflammation, the CD11c+ cells demonstrate an activated phenotype with a 10-100-fold increase in MHCII expression [26], [27], [28]. However, as shown in Figure 1B, the decrease in the abundance of CD11c+ cells in the BAL of IL-19-/- 129xBL6 mice was not associated with an increase in MHCII expression, suggesting a unique phenotype of airway CD11c+ cells in IL-19-/- mice. To understand the nature of the remaining cells in the BAL samples of IL-19-/- 129xBL6 mice, marker-negative cells were purified by fluorescent activated cell sorting and studied by electron microscopy. Cells from the BAL of IL-19KO mice were eosinophils (Fig. 1C); only debris was harvested from the BAL of wild type mice. These cells were not degranulated. To determine if the eosinophils were indicators of tissue inflammation, the lungs of naïve IL-19-/- 129xBL6 mice were examined by histology. No significant inflammation was detected.

### Endogenous IL-19 and strain background determine the abundance of CD11c+ cells in the airways

**Figure 2:**
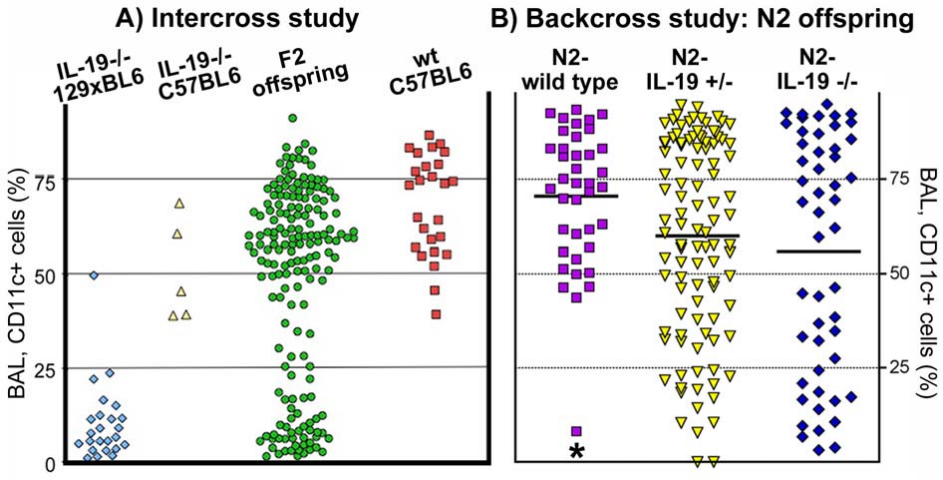
Strain-dependent decreased abundance of CD11c+ cells in the BAL of naive IL-19-/- 129xBL6 mice. The abundance of CD11c+ cells in the BAL (%) from individual mice is shown for the following parental and offspring strains: (**A**) IL-19-/- 129xBL6 (blue diamonds); IL-19-/- C57BL/6 (yellow upright triangles); second generation, intercross F2 offspring from a cross between IL-19-/- 129xBL6 and IL-19-/- C57BL/6 parental lines (green circles); and for comparison, wild-type C57BL/6 (red squares). (**B**) Backcross N2 offspring originating from a cross between IL-19-/- 129xBL6 and wild-type C57BL/6 mice. The mice were genotyped and found to be wild type (*il19* ^+^/*il19* ^+^ purple squares), heterozygous (*il19* ^+^/*il19* ^-^ yellow downward triangles) or IL-19-/- (*il19* ^-^/*il19* ^-^ blue diamonds). Data from individual mice are shown. Horizontal lines indicate means. Data were pooled from 4-6 experiments. Statistical analysis was with ANOVA followed by Dunnett multiple comparison test, * p<0.05 for comparison between N2-wild type and N2-IL-19 -/-.

IL-19-/- mice were backcrossed to C57BL/6 or BALB/c for 6 to 10 times, respectively (Figure S1). In these congenic IL-19-/- mice, the abundance of CD11c+ cells in the BAL was similar to wild-type (Fig. 2A). To determine if the decreased abundance of CD11c+ cells in the BAL of IL-19-/- 129xBL6 mice was due to the environmental microbial flora, the deficiency in IL-19, or a trait from the 129 mouse strain, we intercrossed IL-19-/- 129xBL6 and IL-19-/- C57BL6 mice and we back-crossed IL-19-/- 129xBL6 mice with wild type C57BL/6 mice (Figures S1 and 2). Naïve F2-intercross (Fig. 2A) or N2-backcross (Fig. 2B) offspring was analyzed. Despite the homogeneous environment, the F2-intercross offspring of IL-19-/- 129xBL6 and IL-19-/- C57BL/6 mice had a variable abundance of CD11c+ cells in the BAL (Fig. 2A). Approximately ¼ of the animals demonstrated the characteristic low abundance (<30%) of the CD11c+ cells in the BAL (Fig. 2A). To dissect the inheritance of the phenotype, IL-19-/- 129xBL6 mice were backcrossed to wild-type C57BL/6 mice, the N1 offspring intercrossed, and the N2 offspring analyzed (Fig. 2B). The abundance of CD11c+ cells in the BAL was significantly lower in N2-IL-19-/- mice when compared to N2-wild-type (Fig. 2B, Table S1). Heterozygous N2 offspring also had lower abundance of CD11c+ cells in the BAL relative to wild type (Fig. 2B, Table S1) at a statistically significant level when calculated with the unpaired t test with Welch's correction for unequal variances. As in the IL-19-/- 129xBL6 parent generation, the MHCII expression by the BAL CD11c+ cells remained at wild type levels (data not shown). Taken together, the data shown in Figure 2 demonstrated that the decreased abundance of CD11c+ cells in the BAL of IL-19-/- 129xBL6 mice is determined by the deficiency in IL-19 in interaction with a trait inherited from the 129 mouse strain.

### Endogenous IL-19 regulates the responses of CD11c+ cells to airway challenges with microbial products and pro-inflammatory cytokine

**Figure 3:**
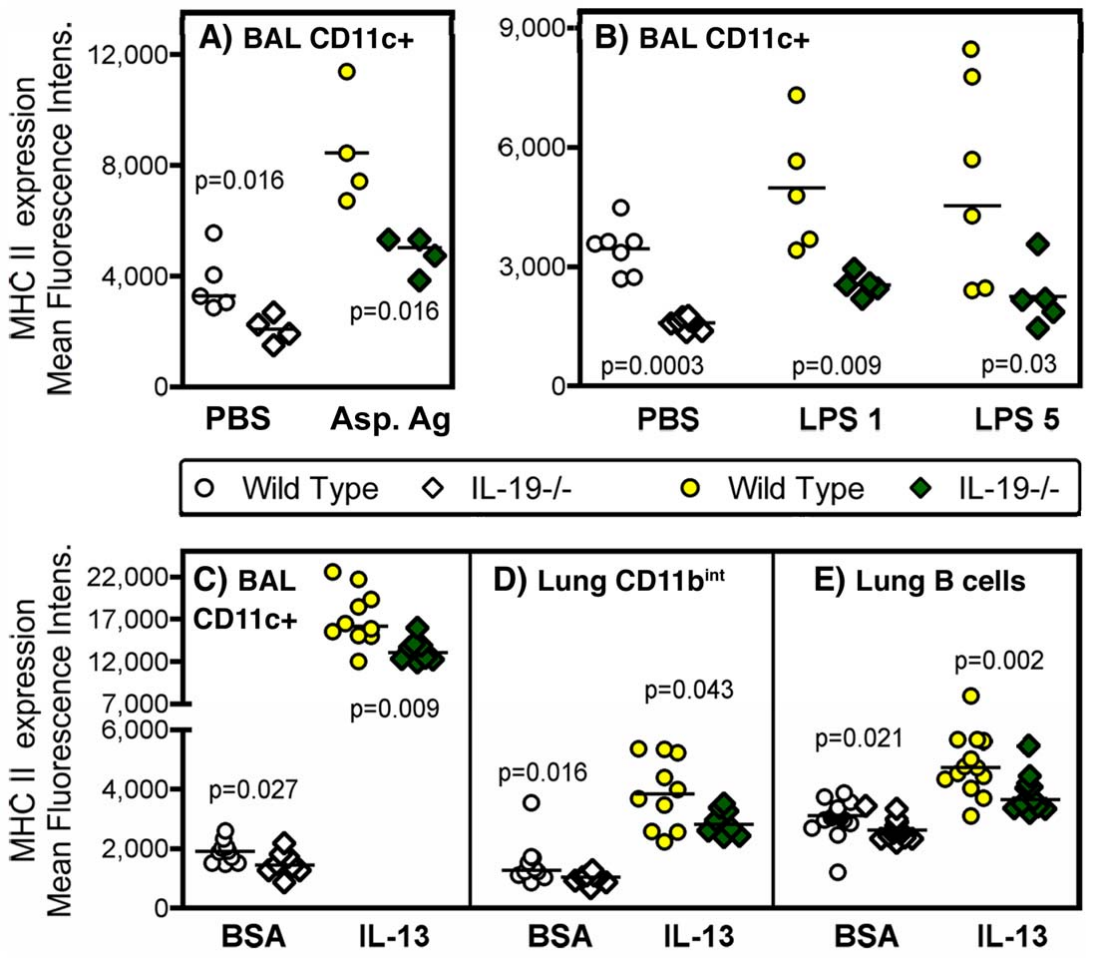
Decreased expression of MHCII in antigen presenting cells from the lungs of IL-19-/- mice. MHCII expression was determined by flow cytometry in CD11c+ cells from the BAL (**A-C**), in CD11b^int^ cells from the lungs (**D**), or in B cells (CD19+, B220+) from the lungs (**E**). The gating strategy is shown in Figure S2. Cells were analyzed from primed animals that were challenged with *Aspergillus* antigen (**A,** Asp. Ag, shaded symbols) and from naïve animals that were challenged with LPS (**B**, at 1 or 5 µg/dose as indicated, shaded symbols) or IL-13 (**C-E**, shaded symbols). Control animals (open symbols) were given saline (PBS) intranasally or saline containing bovine serum albumin (BSA), the carrier protein used to stabilize IL-13. The experimental schedules are outlined in Figure S1. Wild type and IL-19-/- mice were of the BALB/c (**A**), or C57BL/6 (**B-E**) strains. Points represent data for mean fluorescence intensity measured in cells that were harvested from individual wild type (circles), or IL-19-/- (diamonds) mice pooled from 2 experiments each. Horizontal lines indicate medians. Significance levels were calculated with the Mann-Whitney-U test.

The population of CD11c+ cells residing in the airways and lungs is known for their responses to microbial products that result in a 10-100-fold increase in MHCII expression [26], [27], [28] as illustrated in Figure S2. To further understand the role of IL-19 in regulating responses of CD11c+ cells in the lungs, fully backcrossed IL-19-/- and wild type mice were studied following priming and intranasal challenge with the fungal *Aspergillus* antigen extract. In wild-type mice we saw the expected shift (30, 33, 35) in the CD11c+ cell population characterized by very high expression of MHCII (Fig. 3A, 3B). This increase in MHCII expression by CD11c+ BAL cells was significantly ameliorated in antigen-challenged IL-19-/- mice (Fig. 3A, 3B). Similar observations were made in naive IL-19-/- and wild type mice that were intranasally challenged with the bacterial cell wall component lipopolysaccharide, or the pro-allergic Th2 cytokine IL-13 (Fig. 3B, 3C). In all three experimental conditions, BAL CD11c+ cells showed significant reduced MHCII expression at baseline in IL-19-/- mice relative to wild type (Fig. 3A-C). This prompted us to study other MHCII positive cell types that reside in the lungs: monocytes/macrophages and B cells (characterized by the CD11b, CD19 and CD45R-B220 markers, respectively). These cell types also demonstrated decreased MHCII expression at baseline and following intranasal challenge with IL-13 in IL-19-/- mice relative to wild type (Fig. 3D, 3E).

### Endogenous IL-19 regulates Notch2 expression by lung monocytes

**Figure 4:**
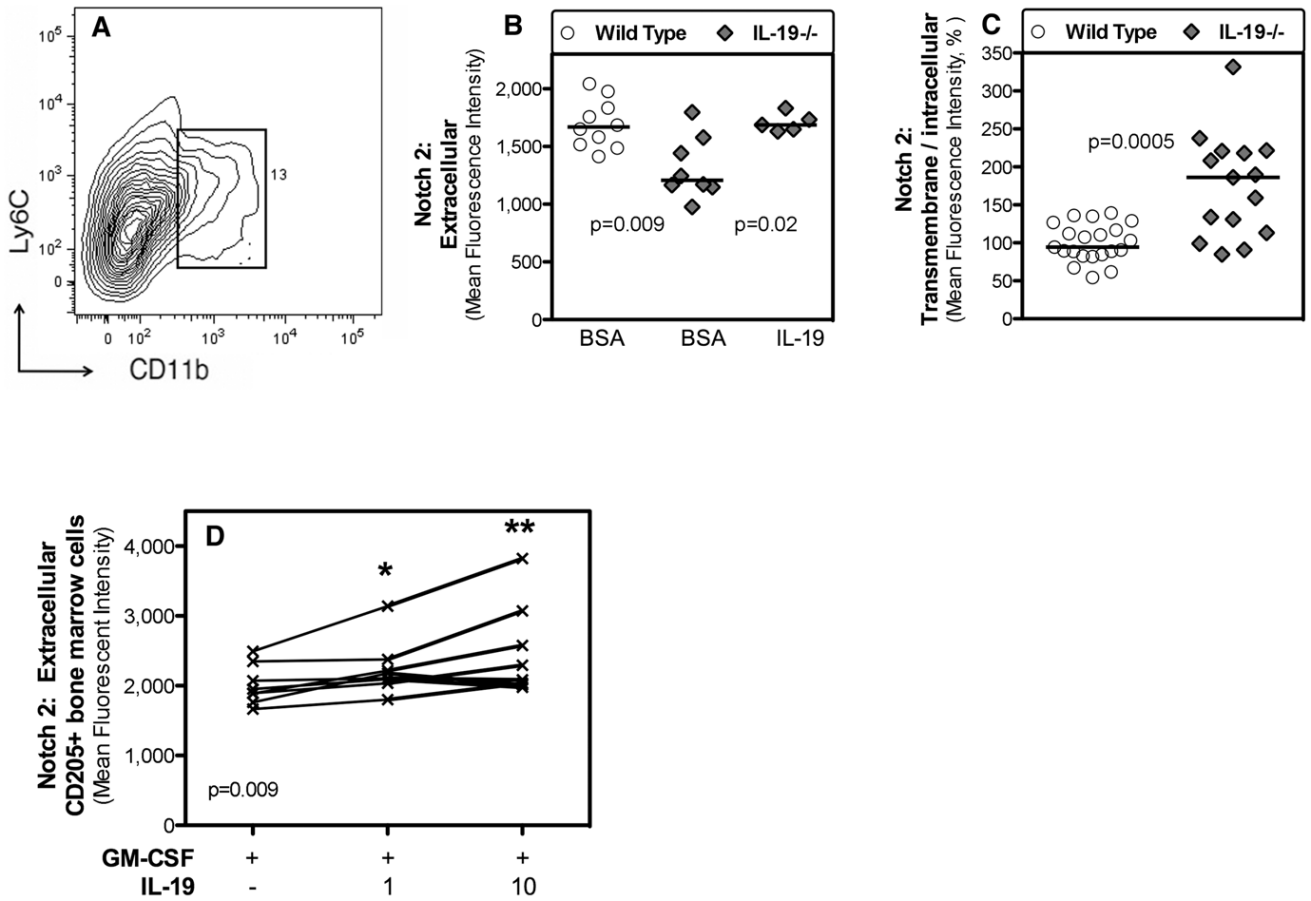
IL-19 regulates Notch2 expression in lung monocytes and bone marrow derived dendritic cells. (**A**) The density plot of lung cells shows the electronic gate used to identify CD11b^int^/Ly6C^int^ lung monocytes. Extracellular (**B**) or transmembrane/intracellular (**C**) Notch2 expression (mean fluorescence intensity) by lung CD11b^int^/Ly6C^int^ monocytes is shown from groups of individual wild type (circles) and IL-19-/- (gray diamonds) mice. The data were pooled from two independent experiments. Horizontal lines indicate medians. (**B**) The mice were given intranasally control protein (bovine serum albumin, BSA) solution or IL-19 (Figure S1). (**C**) Naïve mice were analyzed. The two experiments had different baseline mean fluorescence intensity for Notch2 expression. Therefore, mean fluorescence intensities were plotted relative to the respective median value of wild type mice for each of the experiments and then the data were pooled. (**D**) Extracellular Notch2 expression by bone marrow derived CD205+ dendritic cells. Bone marrow cells were isolated from 8 individual IL-19-/- mice and cultured in the presence of granulocyte-macrophage colony stimulating factor (GM-CSF), with or without the addition of IL-19 at 1 or 10 ng/ml as indicated. CD205+ cells were electronically gated and Notch2 expression was determined by flow cytometry. The lines connect the data from each individual mouse. The data were pooled from two independent experiments. Statistical analysis was with the Friedman test for non-parametric repeated measures (overall p = 0.0009) and Wilcoxon matched-pairs signed rank test (* p<0.01).

To identify a potential mechanism by which IL-19 regulates MHCII expression by CD11c+ cells in the airways, monocytes/macrophages in the lungs and lung B cells, we studied the expression of Notch proteins by lung monocytes (CD11b, Ly6C^intermediate^ cells, Fig. 4A). Notch signaling has been shown to be a critical regulator of immune cell fate and immune homeostasis. Lung monocytes are thought to be precursors of CD11c+ cells in the airways in the resting state (36). Using antibodies that recognize different epitopes on the Notch2 molecule, we found that lung monocytes from IL-19-/- C57BL/6 mice expressed extracellular Notch2 at significantly lower levels relative to wild-type (Fig. 4B). Intranasal administration of recombinant IL-19 to IL-19-/- mice resulted in significantly increased extracellular Notch2 expression by lung monocytes (Fig. 4B). In contrast, transmembrane/intracellular Notch 2 expression was significantly increased in IL-19-/- C57BL/6 mice (Fig. 4C).

### Bone marrow derived dendritic cells (DCs) cultured in the presence of IL-19 expressed significantly higher levels of extracellular Notch2

Under inflammatory conditions, airway and lung CD11c+ cells are known to be replenished from migrating bone marrow progenitor cells [29], [30], [31] and to have a DC phenotype characterized by high levels of MHCII. Therefore, we cultured lineage-depleted bone marrow cells harvested from IL-19-/- mice in the presence of granulocyte-macrophage colony stimulating factor (GM-CSF). These culture conditions are known to expand and differentiate DCs characterized by expression of CD11c and CD205. We measured extracellular Notch2 expression by DCs that were identified by the CD205 marker. We found that the presence of IL-19 in the cultures significantly increased extracellular Notch2 expression (Fig. 4D).

### Abnormal CD11c+ cell compartment in the airways and lungs of naïve IL-19-/- C57BL/6 mice

**Figure 5:**
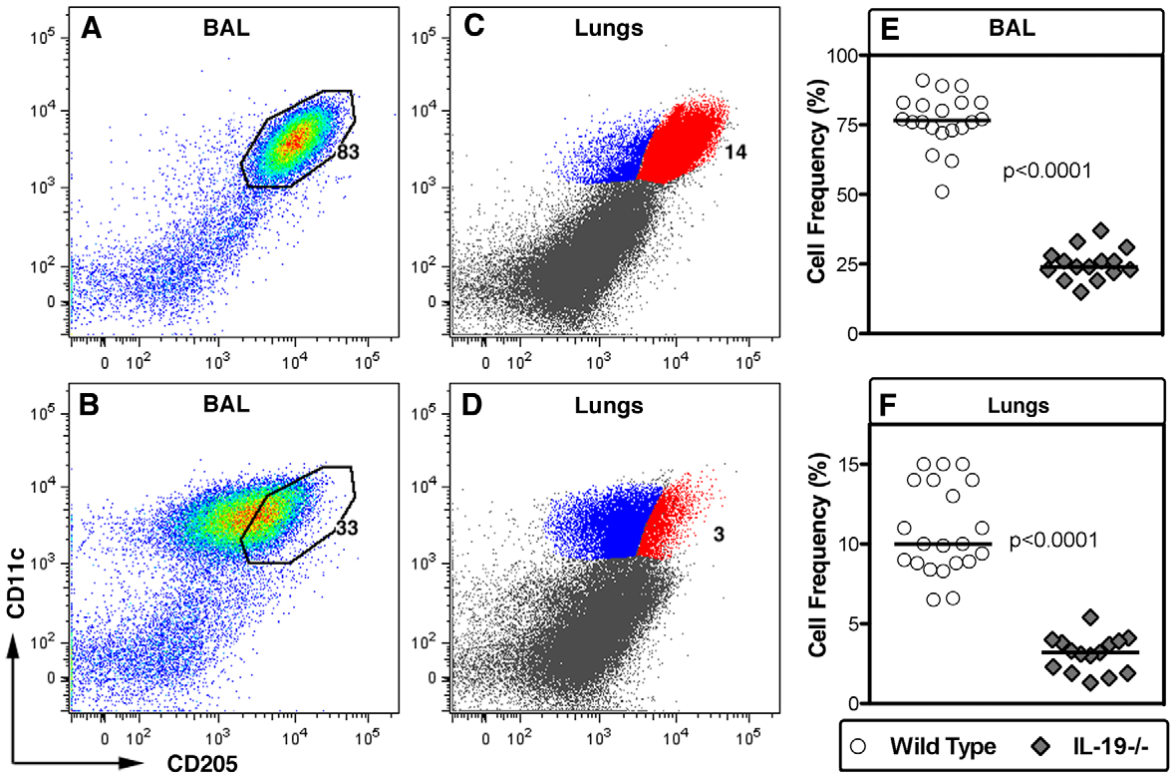
IL-19 regulates the relative size of the CD205+ subset of CD11c+ cells in the airways and lungs of naïve mice. (**A-D**) The dot plots show CD11c (y-axis) vs. CD205 (x-axis) staining of BAL (**A, B**), and lung (**C, D**) cells. Representative plots from naïve wild type (**A, C**, n = 21) and IL-19-/- (**B, D**, n = 16) C57BL/6 mice are shown. The percentage of cells that express CD11c and CD205 at high levels is indicated. (**E, F**) Group comparisons of naïve C57BL/6 wild type (circles) and IL-19-/- (gray diamonds) mice for percentages of CD11c+ CD205+ cells in the BAL (**E**) or in cell suspensions prepared from the lung parenchyma (**F**). Individual data points that were pooled from two experiments are shown. Horizontal lines indicate medians. Significance levels were calculated with the Mann-Whitney-U test**.** Note that the numbers of CD11c+ cells in the BAL and lungs of wild type and IL-19-/- mice were similar (Table S2).

All data thus far indicated that IL-19 regulates CD11c+ cells in the airways and lungs at baseline and the behavior of these cells following inflammatory challenge. However, in C57BL/6 strain mice, there was no difference between wild type and IL-19-/- in the numbers of CD11c+ cells in the BAL or lungs (Table S2). To understand these data better, the CD11c+ cells were studied for the expression of CD205, an important DC subset marker [32], [33], [34]. In wild type mice, the majority of the CD11c+ cells from BAL or lungs co-express CD205 at high levels (Fig. 5A, 5C). In IL-19-/- C57BL6 mice, however, the fraction of CD11c+ cells in the BAL or lungs co-expressing CD205 was reduced by 3-5 fold (Fig. 5B, 5D-F). Data of wild type and IL-19-/- C57BL/6 mice did not show overlap with respect to the frequency of CD11c+CD205+ cells in the BAL or lungs (Fig. 5E, 5F).

### Endogenous IL-19 regulates inflammation and histologic changes in response to a T cell-dependent-fungal antigen, but not T cell-independent pro-inflammatory agents

**Figure 6:**
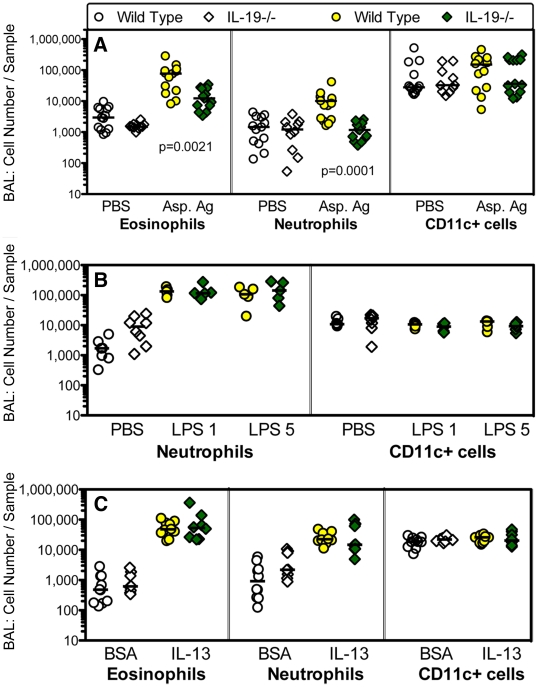
IL-19-/- mice have a decreased inflammatory response to a T cell-dependent challenge of the airways. (**A**) Groups of IL-19-/- (diamonds) and wild type (circles) mice were primed with *Aspergillus* antigen and challenged with antigen intranasally (Asp. Ag, shaded symbols). (**B, C**) Groups of naïve mice were given lipopolysaccharide (**B**, LPS, 1 or 5 µg/dose as indicated) or IL-13 (**C**) intranasally shown as shaded symbols. Control animals received saline (PBS) or control protein (bovine serum albumin, BSA) intranasally (**A-C**, open symbols). Figure S1 shows the experimental schedules; Figure S2 the technique for the determination of eosinophils, neutrophils, and CD11c+ cells in the BAL by flow cytometry. IL-19-/- and wild type mice were of the BALB/c (**A**), or C57BL/6 (**B, C**) background strains. Groups of mice were pooled from 2-3 independent experiments. Individual data are shown. Horizontal lines represent medians. Significance levels were calculated with the Mann Whitney U test.

**Figure 7:**
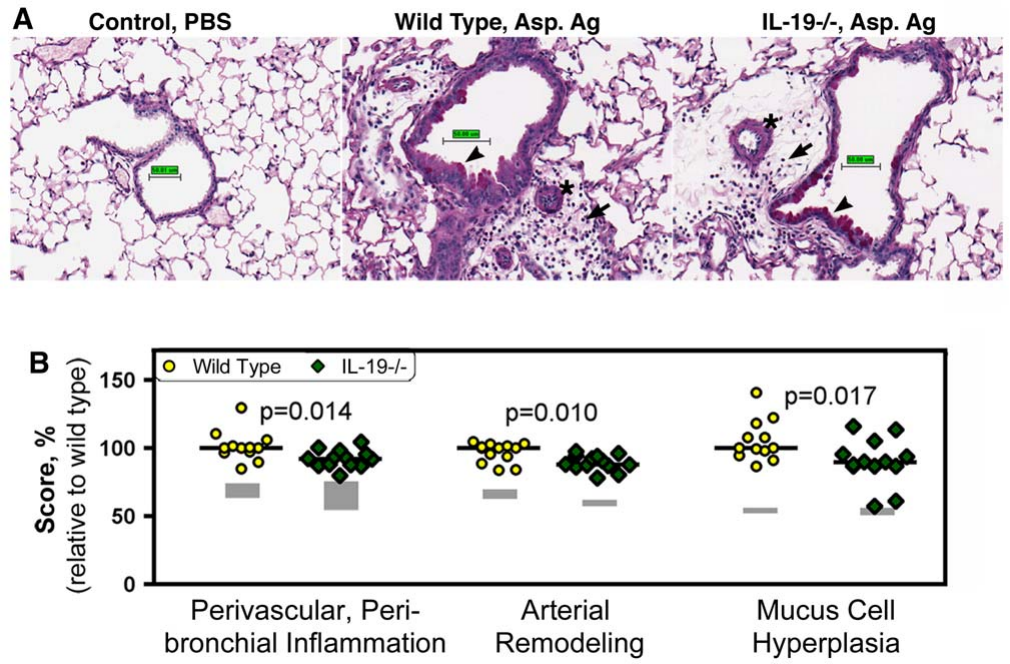
Histological changes in the lungs of wild type and IL-19-/- mice primed and challenged with Aspergillus fumigatus antigen. Wild type and IL-19-/- mice were of the BALB/c background strain. Priming and challenge with *Aspergillus* antigen was as indicated in Figure S1. Control animals were given saline (PBS) intranasally. (**A**) Photomicrographs of lung sections from wild type or IL-19-/- mice. The sections were stained with periodic acid schiff and digitally scanned. The software was used to generate the scale bar (50 µm). Perivascular/peribronchial inflammation (arrows), pulmonary arterial remodeling (stars) and mucus cell hyperplasia (arrow heads) are indicated. (**B**) Scores for perivascular/peribronchial inflammation, arterial remodeling and mucus cell hyperplasia in the lungs of wild-type (circles) or IL-19-/- (diamonds) mice. The gray boxes outline the values from the groups of control animals depicting the scores spanning the 25% and the 75% quartiles. Dots represent individual data from *Aspergillus* antigen primed and challenged mice. Horizontal lines show medians. Three independent experiments were performed during a time span of 6 years. Therefore, scores needed to be standardized. For this reason, scores were plotted relative to the respective median scores of primed and antigen-challenged wild-type mice for each experiment and then the data were pooled. Significance levels were calculated with the Mann-Whitney-U test.

Because MHCII is the molecular platform that presents antigen-derived peptides to T cells, we studied T cell-dependent and T cell independent airway inflammation and tissue remodeling changes in the lungs. T cell-dependent responses were induced by priming and intranasal challenge with Aspergillus antigen (Fig. S1A). T cell-independent responses were elicited by the administration of lipopolysaccharide or recombinant IL-13 to naive mice (Fig. S1B, S1C). We found that the T cell-dependent challenge induced inflammatory infiltrates into the airways measured by the analysis of BAL and histological changes in the lungs that were significantly ameliorated in IL-19-/- mice relative to wild type (Fig. 6A, Fig. 7). In contrast, airway inflammation elicited by the T cell-independent challenges was similar in IL-19-/- and wild type mice (Fig. 6B, 6C).

## Discussion

IL-19 is a member of the IL-10 family of cytokines [15], [35]. As such, one of the expectations of our studies was that IL-19 would have anti-inflammatory effects, just like IL-10. The notion that IL-19 can have anti-inflammatory activity has been supported by recent publications from two groups whose work has focused on innate immune responses in the colon and responses of vascular smooth muscle and endothelial cells, respectively [36], [37], [38], [39], [40].

Our studies argue against these expectations in the lungs and, instead, suggest that endogenous IL-19 is a constituent of the regulome that controls homeostasis in the lungs with respect to MHCII and Notch2 cell surface expression. In keeping with the function of MHCII in presenting antigenic peptides to T helper cells, the decreased MHCII expression in several cell types in the lungs of IL-19-/- mice was associated with a significantly ameliorated response to intranasal challenge with a T cell-dependent microbial antigen, *Aspergillus* antigen. In contrast, our studies did not reveal increased or decreased inflammatory changes in IL-19-/- mice intranasally challenged with the microbial product lipopolysaccharide or the pro-allergic cytokine IL-13, both of which induce T cell-independent inflammation. Different routes of innate challenge, lung (in our studies) or colon (in the studies reported by Dr. Azuma and colleagues [39], [40]), might have contributed to the apparent difference in the biological role of IL-19.

IL-19 has been implicated in the pathogenesis of human asthma by increased levels of this cytokine in the serum of patients [1]. IL-19 has been shown to increase Th2 cytokine production [1], [41], but mice deficient in the IL-20RB component of the IL-19 receptor showed increased cytokine elaboration, particularly of the Th1 type [42]. The latter results could be due to competitive receptor use by IL-20 and IL-24 that share the IL-20RA/IL-20RB with IL-19, and that also signal through the IL-22R/IL-20RB receptor complex, which does not bind IL-19 [18], [42]. While IL-19 has been reported to stimulate the production of IL-10 [43], our data did not indicate a deficiency of IL-10 production in IL-19-/- mice since we did not see an increase in the sensitivity to lipopolysaccharide that is typical for the absence of IL-10 [44], [45].

Our studies demonstrated a critical role of endogenous IL-19 in determining the abundance of CD11c+ cells in naïve 129xBL6 mice. Backcross and intercross experiments demonstrated that this phenotype was due to the combination of the deficiency in IL-19 and a trait inherited from the 129-mouse strain. This trait encompassing one or more genes remains to be identified. Careful examination of CD11c+ cells harvested from the airways or from the lung parenchyma of C57BL/6 mice revealed a large (3-5 fold) reduction in the subset that expresses CD205 at high levels. While CD11c+CD205+ spleen and lymph node DCs are known to be functionally distinct from CD11c+CD205- DCs [32], [33], [34], the segregation of function in the airways and lungs between the subsets of CD11+ cells expressing high or low levels of CD205 remains to be identified. Together, our findings emphasize the significance of IL-19 in the regulome that controls the homeostasis of CD11c+ cells in the airways and lungs *in vivo*. Our data are supported by *in vitro* studies showing that IL-19 induces monocytes to produce innate cytokines, reactive oxygen species [46] and auto-induces its own production [43].

Naïve IL-19-/- C57BL/6 mice that were challenged with IL-13 intranasally showed decreased MHCII expression by lung B cells. This observation could be related to the decreased expression of extracellular Notch2 and the increased expression of transmembrane/intracellular Notch2 by lung monocytes in naïve IL-19-/- mice relative to wild-type. Notch2 is a very large molecule with complex structure that has an extracellular receptor module and an intracellular nuclear signaling module. Future experiments will need to address the question of whether IL-19 determines the extracellular receptor-ligand binding activity, the intracellular signaling activity or both functions of the Notch2 molecule. Notch2 is a receptor that provides essential signals for the development of specific B cell subsets [47], [48], [49]. While the role of IL-19 in determining the expression of Notch receptors in B cells remains to be determined, our data strengthen the notion that one of the biological roles of IL-19 is to regulate the stimulation of B cells [50] and B cell responses in asthma [1].

Our data demonstrate that endogenous IL-19 is a constituent of the regulome that controls the responses of (a) BAL CD11c+ cells enhancing cell surface MHCII and CD205 expression, (b) lung monocytes regulating Notch2 expression, and that (c) exacerbates airway inflammation and histological changes in the lungs to T-cell dependent antigen challenge. Airway epithelial cells might amplify this pro-immunogenic activity of IL-19 because both human and mouse bronchial epithelial cells show increased production of IL-19 following stimulation with the Th2 cytokines IL-4 and IL-13 [3], [51] and IL-19 stimulation of Th2 cells results in increased production of Th2 cytokines [1], [41], [52].

Our study, together with previously published reports detailing the significance of IL-19 in infectious [20], [53], [54], allergic [1], [2], [3], [4] and autoimmune diseases [5], [6], [7], [8], [9], [10], [11], [12], [13] suggests a potential clinical application. Recombinant IL-19 or IL-19 inhibitors might be useful clinically in instances that call for modulation of MHCII expressing cells and of CD11c+ CD205+ cells in the lungs.

## Materials and Methods

### Ethics Statement

All animal experiments were performed according to guidelines outlined by the United States Department of Agriculture and the American Association of Laboratory Animal Care under the supervision and specific approval of the Institutional Animal Care and Use Committees at St. Luke's Roosevelt Hospital (IACUC numbers GR0205, GR0206), Columbia University (IACUC number AC-AAAA7912) and New York University (IACUC number 081114, most recent approval date: 11/17/2010) (New York, NY). The named IACUC committees specifically approved this study.

### Mice

The IL-19-/- mice on the 129xBL6 background were previously described [55]. The mice were speed-backcrossed to the C57BL/6 for 6 generations or backcrossed to the BALB/c strain for 11 generations. Wild type C57BL/6 and BALBc mice were purchased from the Jackson Laboratory. To determine environmental or genetic trait influences on the function of endogenous IL-19, F2 and N2 mice were studied. F2 mice were generated by crossing IL-19-/- 129xBL6 mice with IL-19-/- C57BL/6 mice followed by intercrossing the F1 offspring. N2 mice were made by crossing IL-19-/- 129xBL6 mice with wild type C57BL/6 mice followed by intercrossing the N1 offspring. The mouse strains are listed in Figure S1.

The mice were housed in filter-top cages under specific pathogen-free conditions at St. Luke's Roosevelt Hospital, New York, New York, Charles River Laboratories, Wilmington, Massachusetts, or the Institute for Environmental Medicine, New York University, Tuxedo, New York. All experiments were performed according to guidelines outlined by the United States Department of Agriculture and the American Association of Laboratory Animal Care under the supervision of the Institutional Animal Care and Use Committees at St. Luke's Roosevelt Hospital, Columbia University or New York University (New York, New York).

### BAL cell analysis by electron microscopy

Marker negative cells were purified by sorting using an Aria fluorescent activated cell sorter (Becton Dickinson) from a pool of BAL cells harvested from 20 to 25 mice in each group of naïve wild type 129xBL6 mice or IL-19-/- 129xBL6 mice. The cells were fixed and photographed via electron microscopy at the Rockefeller University Microscopy Core facility.

### Allergen exposure

Groups of mice were either given phosphate buffered saline (PBS) or primed and intranasally challenged with Aspergillus antigen known to induce T cell-dependent airway inflammation and lung tissue remodeling [26], [44], [56]. Priming with Aspergillus antigen (crude antigen free of viable fungus) consisted of three weekly intraperitoneal injections of 100 µg Aspergillus antigen in a 100-µl volume of PBS (Fig. S1A). One week later, challenges were given intranasally to lightly anaesthetized mice with 100 µg of antigen in a 50-µl volume of PBS once. Control groups were given PBS intraperitoneally and intranasally. The mice were euthanized 4 days after the final intranasal exposure to Aspergillus antigen because our previous work has shown that this is the optimal time point to assess inflammation and pathologic changes in the lungs [44].

### Lipopolysaccharide

Naive wild-type or IL-19-/- mice were administered lipopolysaccharide (Sigma-Aldrich, Minneapolis, Minnesota), or PBS once as outlined in Figure S1B. Lipopolysaccharide was diluted in phosphate buffered saline and administered intranasally at doses of 1 or 5 µg in a 50-µl volume to lightly anaesthetized naïve mice. The animals were euthanized one day later.

### Recombinant Cytokines

Naive wild-type or IL-19-/- mice were administered recombinant mouse IL-19 (R&D Systems, Minneapolis, MN), or IL-13 (Peprotech) diluted in PBS solution containing bovine serum albumin, or PBS solution containing bovine serum albumin only (bovine serum albumin, low in lipopolysaccharide; 2.5 mg/ml; Sigma-Aldrich) for 3 times at two-day intervals. The cytokines were administered intranasally at a dose of 5 µg in a 50-µl volume to lightly anaesthetized naïve mice as outlined in Figure S1C [24], [26], [56]. The animals were euthanized one day following the last administration of cytokine.

### Tissue recovery

At the end of each experiment, the mice were euthanized by an overdose of barbiturate and tissues were harvested [24], [26], [56]. BAL was performed by 3 instillations of 1 ml of Hanks balanced salt solution that was gently retrieved. The left lung lobe was sutured at the bronchial stem and removed into Hanks balanced salt solution. A single cell suspension was prepared by gently mushing the lung tissue using the plunger of a syringe and a cell strainer, followed by straining through nylon-mesh cell strainers. The remaining lung lobes were inflated with 0.5 ml of 10% formaldehyde buffered with PBS and removed into formaldehyde.

### Histology

Sections of lungs were stained with hematoxylin and eosin (H&E) or Periodic Acid Schiff (PAS). The sections were examined under 200x or 400x magnification. Histological changes were scored by examining 20-40 consecutive view fields as described before [24], [26], [56]. Briefly, the scores for *peribronchia/perivascular inflammation* were: (0) normal with very few inflammatory cells; (1) scattered inflammatory cells up to two rings in depth; (2) cuffs of inflammatory cells measuring three rings or more in depth; or (3) dense ring of inflammatory cells. *Interstitial inflammation* was scored as: (0) normal; (1) increased numbers of cells within the alveoli; or (2) consistent increase in the numbers of cells within the alveoli, appearance of multinucleated giant cells, and thickening of the alveolar septa. *Pulmonary arterial remodeling* was scored as: (0) normal; (1) up to a doubling of the depth vascular wall with intact lumen and circular media (all cells follow the form given by the endothelium); (2) thickening of the vascular wall by more than 3 x than normal; or (3) lumen appears to be obstructed and the wall is thickened and lined with disorganized layers of cells (cells in the blood vessel wall assume a pattern that differs from the lumen). *Mucus cell hyperplasia* was scored as: (0) normal; (1) less than 30% of the epithelial cells; (2) >30–70%; or (3) > 70% of the cells lining the airway lumen were stained with the periodic acid schiff reagent.

Slides were scanned with the Aperio slide scanner to capture digital images. Aperio software was used that is available for free download from the aperio website (http://www.aperio.com/download-imagescope-viewer.asp). Scale bars were generated with the Aperio software.

### Flow Cytometry

Bronchoalveolar lavage (BAL) cells and cell suspensions prepared from the lung lobes were analyzed by flow cytometry as described [26], [44], [56], [57] using BD Calibur (BD Biosciences) and MACS Quant (Miltenyi Biotechnology) instruments and FloJo (TreeStar Inc) software (Fig. S2).

BAL samples were studied (Fig. S2) for the presence of eosinophils (CD11b^high^, CCR3^high^, GR1^low^, CD11c^low-intermediate^, MHCII^low-intermediate^), neutrophils (CD11b^high^, GR1^high^, CD11c^low-intermediate^, MHCII^low-intermediate^), and CD11c+ cells. CD11c+ cells were defined in the dot plots depicting CD11c+ staining versus side scatter. The CD205+ subset of CD11c+ cells was identified by staining with the Dec205 monoclonal antibody tagged with PeCy7 (Biolegend). Dendritic cells (DCs) were identified as a subpopulation of the CD11c+ cells that was MHC II^high^ and the majority of these cells also expressed CD11b at high levels [26], [27], [28], [58]. To verify the data obtained by flow cytometry, aliquots of the BAL samples were spun onto slides using a cytocentrifuge, stained with Wrightes/Giemsa and analyzed by microscopy.

Lung cell suspensions (Fig. S2) were studied for CD11c+ cells, monocytes/macrophages (CD11b^intermediate^, CCR3^low^, MHC II^low^), monocytes (CD11b^intermediate^, Ly6C^intermediate^) and B cells (CD45R/B220^+^ and CD19^+^).

The cell populations were analyzed, as shown in Figure S2, using monoclonal antibodies tagged with Pacific Blue, AlexaFluor, fluorescein (FITC), phycoerythrin (PE), peridinin-chlorophyll (PerCP), Allophycocyanin (APC), or cyanine (Cy) tandem dyes that were purchased from BD Bioscience (San Jose, California), Ebioscience (San Diego, California) or Biolegend (San Diego, California) as follows: anti-CD11c (clone N418) tagged with PE, APC, or Pacific Blue; anti-CD11b (clone M1/70) tagged with PE or APC; anti-MHCII (I-A/I-E, clone M5/114.15.2) tagged with FITC or biotin (detected with Streptavidin-PerCP); anti-Ly-6G/Ly-6C (clone GR1) tagged with biotin (detected with Streptavidin-PerCP) or APC-Cy7; anti-F4/80 (clone 8M8) tagged with APC; anti-CCR3 (clone TG14, Biolegend, or clone 83103, BD Bioscience) tagged with AlexaFluor 647.

Expression of Notch2 was analyzed with antibodies detecting different epitopes of Notch 2. Extracellular Notch2: hamster anti-mouse Notch2 (clone HMN2-35, Biolegend) purified, detected with biotinylated anti-Armenian Hamster and Streptavidin-PerCP; or rat anti mouse/human Notch 2 (clone 16F11, Ebioscience) tagged with PE. Transmembrane/intracellular Notch2: rabbit monoclonal anti mouse/human Notch 2 (clone D76A6, Cell Signaling Technology, Danvers, Massachusetts) purified, detected with APC labeled donkey anti-rabbit multi-absorbed antibody (Fab2 fragment, Jackson ImmunoResearch, West Grove, Pennsylvania).

B cells were detected in the lungs using anti-CD45R/B220 (clone RA3-6B2) tagged with Pacific Blue or PerCP and anti-CD19 (clone 6D5) tagged with PE-Cy7 or APC.

### Bone marrow cultures

Bone marrow cells were harvested from one femur per mouse and depleted of lineage + cells using anti-Ter119, CD11b, B220, CD5, GR1 antibodies and magnetic beads. The cells were harvested and seeded into 96 well plates at a cell density of 50,000 -100,000 cells in a total volume of 200 µl per well. The cell culture medium was RPMI1640 supplemented with Penicillin (100 U), Streptomycin (100 µg), Hepes buffer (10 mM), Sodium-pyruvate (1 mM), L-Glutamine (2 mM), ß2 mercapto-ethanol (10 µM) and 10% fetal calf serum. The fetal calf serum lot was selected for low lipopolysaccharide content. The cells of each mouse were cultured with granulocyte-macrophage colony stimulating factor (GM-CSF) at 5 ng/ml, with or without IL-19 at 1 or 10 ng/ml. The cultures were performed in duplicate. The cells were incubated for 7 days in humidified atmosphere in 5% CO2. The cells were then directly stained in the wells with anti–CD205 and anti-Notch2 antibodies and read by flow cytometry. The cells were gated for CD205 and the mean fluorescence intensity of extracellular Notch2 was recorded. The mean of the duplicate wells for each sample was calculated and plotted.

### Statistics

Differences between two groups were analyzed using the two-tailed, unpaired Mann-Whitney-U test. Statistical analysis of backcross experiments was with ANOVA followed by Dunnett multiple comparison test. The bone marrow culture study was analyzed with the Friedman test for non-parametric repeated measures and the Wilcoxon matched-pairs signed rank test. The data were analyzed with the GrapPad Prism software. A significant difference between groups was assumed when the p value was 0.05 or lower.

## Supporting Information

Figure S1 **Mouse strains studied and experimental schedules**. List of IL-19-/- mouse strains. Schematic representations of the experimental schedules are shown for (**A**) Aspergillus antigen (Asp. Ag) priming by intraperitoneal (i.p.) injections followed by intranasal (i.n.) challenge, (**B**) lipopolysaccharide (LPS), or (**C**) recombinant cytokine (IL-13 or IL-19) challenge via the intranasal (i.n.) route of naïve mice.
(TIF)

Figure S2 **Gating strategy for the detection of cell populations in the BAL and lungs.** (**A, B**) Gating for CD11c+ cells in the BAL in dot plots demonstrating CD11c staining (Y-axis) and Side Scatter (X-axis) from a naïve mouse (**A**) or a mouse undergoing Th2 inflammation (**B**). (**C**) Dendritic cells (DCs) identified from the CD11c+ population in plots of CD11c (Y-axis) and MHCII (X-axis) staining. The overlay dot plot technique was used to draw a gate that separates CD11c+ cells that express low levels of MHCII (typical for naïve control), or high levels of MHCII (DCs, typically increased in Th2 inflammation). (**D**) Neutrophils and eosinophils identified in a dot plot of BAL cells by the expression of GR1 (Y-axis) and CD11b (X-axis). Neutrophils expressed high levels of GR1 and CD11b; eosinophils low levels of GR1 and high levels of CD11b. (**E**) In neutrophil and eosinophil populations, here shown for eosinophils, DCs were excluded by gating on cells that are CD11c ^low-intermediate^ and MHCII^low to intermediate^. **(F-H)** CD11b^int^ monocytes (CD11b^intermediate^, CCR3^negative-low^) in dot plots showing CCR3 (Y-axis) and CD11b (X-axis) staining of naïve BAL (**F**), naïve lung (**G**) or IL-13 challenged lung (**H**). Eosinophils (CCR3^high^, CD11b^high^) and neutrophils (CCR3^low^, CD11b^high^) were also distinguished in these plots.
(TIF)

Table S1 **Distribution of the abundance of CD11c+ cells in the BAL from N2 offspring mice.**
(DOC)

Table S2 **Numbers of CD11c+ cells in the BAL and lungs and expression of CD205 by CD11c+ cells.**
(DOC)
