# Supplementary figures and images for: Interleukin-19: A Constituent of the Regulome That Controls Antigen Presenting Cells in the Lungs and Airway Responses to Microbial Products
Source: PLoS One. 2011 Nov 15;6(11):e27629. doi: 10.1371/journal.pone.0027629 (PMC3217014; doi:10.1371/journal.pone.0027629)

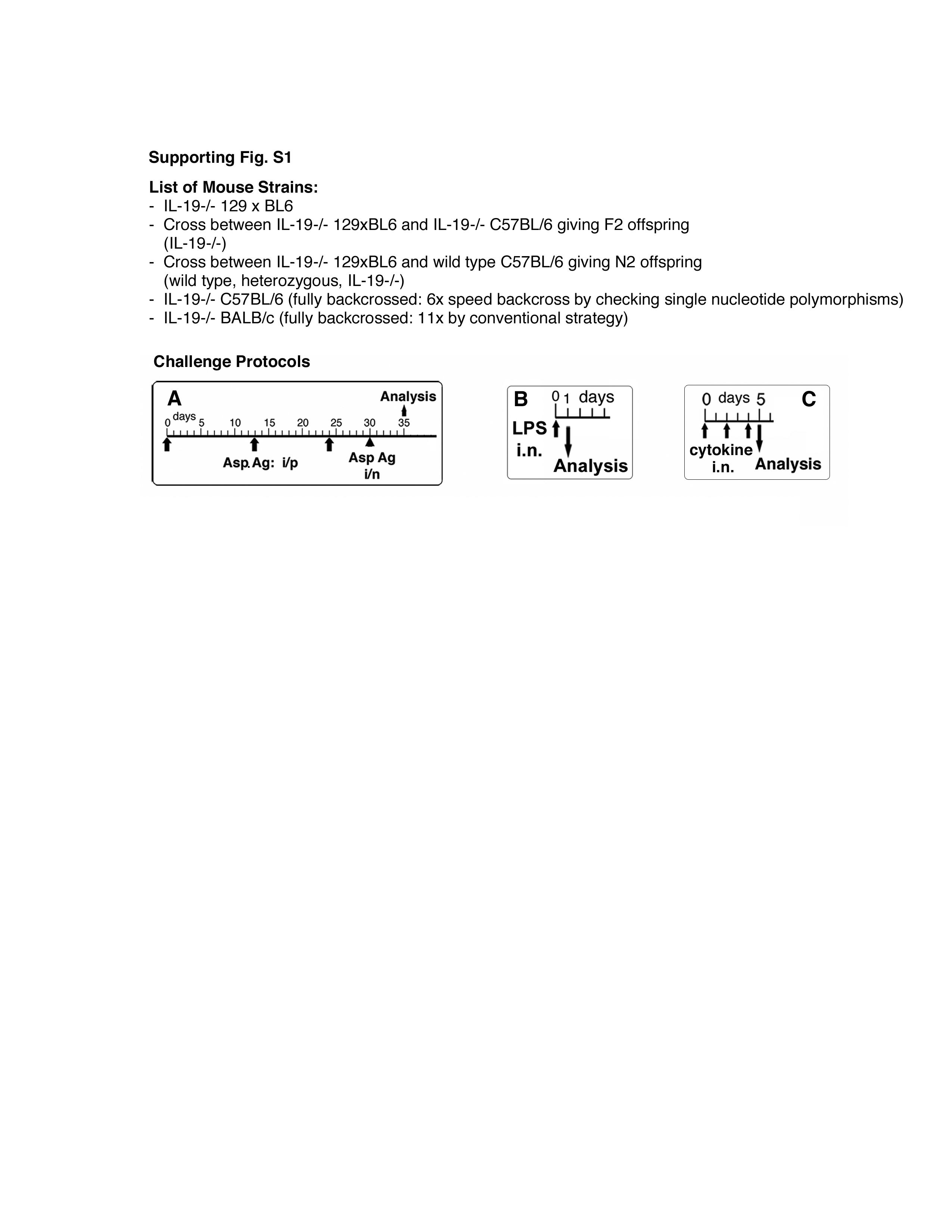

Supplement: Figure S1 — Mouse strains studied and experimental schedules. List of IL-19-/- mouse strains. Schematic representations of the experimental schedules are shown for (A) Aspergillus antigen (Asp. Ag) priming by intraperitoneal (i.p.) injections followed by intranasal (i.n.) challenge, (B) lipopolysaccharide (LPS), or (C) recombinant cytokine (IL-13 or IL-19) challenge via the intranasal (i.n.) route of naïve mice. (TIF) [file pone.0027629.s001.tif]

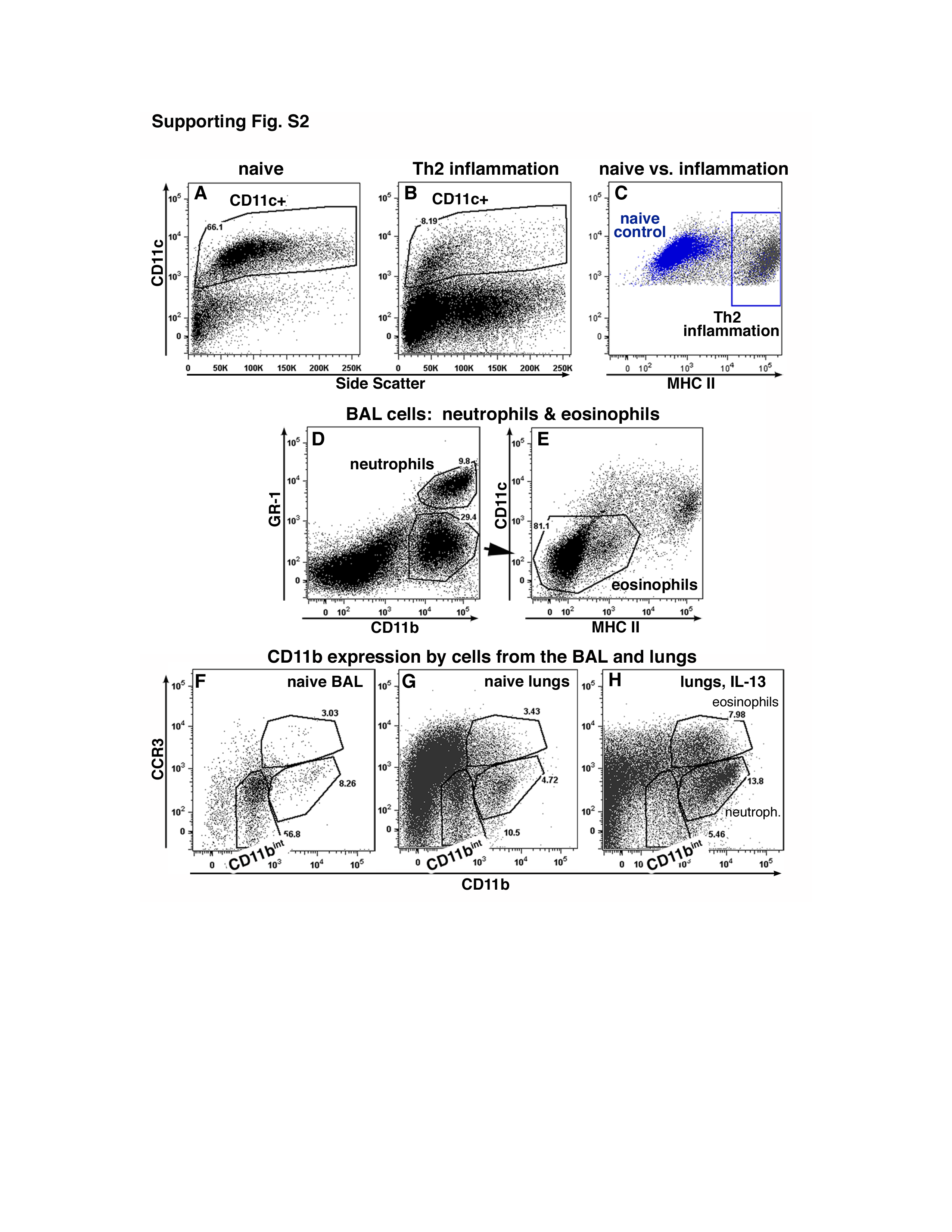

Supplement: Figure S2 — Gating strategy for the detection of cell populations in the BAL and lungs. (A, B) Gating for CD11c+ cells in the BAL in dot plots demonstrating CD11c staining (Y-axis) and Side Scatter (X-axis) from a naïve mouse (A) or a mouse undergoing Th2 inflammation (B). (C) Dendritic cells (DCs) identified from the CD11c+ population in plots of CD11c (Y-axis) and MHCII (X-axis) staining. The overlay dot plot technique was used to draw a gate that separates CD11c+ cells that express low levels of MHCII (typical for naïve control), or high levels of MHCII (DCs, typically increased in Th2 inflammation). (D) Neutrophils and eosinophils identified in a dot plot of BAL cells by the expression of GR1 (Y-axis) and CD11b (X-axis). Neutrophils expressed high levels of GR1 and CD11b; eosinophils low levels of GR1 and high levels of CD11b. (E) In neutrophil and eosinophil populations, here shown for eosinophils, DCs were excluded by gating on cells that are CD11c low-intermediate and MHCIIlow to intermediate. (F-H) CD11bint monocytes (CD11bintermediate, CCR3negative-low) in dot plots showing CCR3 (Y-axis) and CD11b (X-axis) staining of naïve BAL (F), naïve lung (G) or IL-13 challenged lung (H). Eosinophils (CCR3high, CD11bhigh) and neutrophils (CCR3low, CD11bhigh) were also distinguished in these plots. (TIF) [file pone.0027629.s002.tif]
