# Supplementary material for: Interleukin-19: A Constituent of the Regulome That Controls Antigen Presenting Cells in the Lungs and Airway Responses to Microbial Products
Source: PLoS One. 2011 Nov 15;6(11):e27629. doi: 10.1371/journal.pone.0027629 (PMC3217014; doi:10.1371/journal.pone.0027629)
Supplement: Table S1 — Distribution of the abundance of CD11c+ cells in the BAL from N2 offspring mice. (DOC) [file pone.0027629.s003.doc]

**Supporting Table S1: Distribution of the abundance of CD11c+ cells in the BAL from N2 offspring mice**.

| **Abundance of CD11c+ cells in the BAL (%)** | **N2- wild type (+/+)** | **N2- heterozygous (+/-)** | **N2-KO**  **(-/-)** |
| --- | --- | --- | --- |
| **> 50 %** | 35 | 69 | 27 |
| **< 50 %** | 4 | 33 | 23 |
| **Total** | 39 | 102 | 50 |

BAL cells from N2 offspring mice from a backcross between IL-19-/- 129xBL6 and C57BL/6 wild type mice were analyzed by flow cytometry. Statistical analysis of the data distribution among the groups was performed with the Chi-Square test, p<0.005.
