# Supplementary material for: Interleukin-19: A Constituent of the Regulome That Controls Antigen Presenting Cells in the Lungs and Airway Responses to Microbial Products
Source: PLoS One. 2011 Nov 15;6(11):e27629. doi: 10.1371/journal.pone.0027629 (PMC3217014; doi:10.1371/journal.pone.0027629)
Supplement: Table S2 — Numbers of CD11c+ cells in the BAL and lungs and expression of CD205 by CD11c+ cells. (DOC) [file pone.0027629.s004.doc]

**Supporting Table S2: Numbers of CD11c+ cells in the BAL and lungs and expression of CD205 by CD11c+ cells.**

|  | **Wild Type** | **IL-19-/-** |
| --- | --- | --- |
| **Number of CD11c+ cells in the BAL** | 24,565 ± 3,031 | 33,599 ± 3,379 ^ns^ |
| **Number of CD11c+ cells in the lungs** | 67,426 ± 6,988 | 57,479 ± 6,612 ^ns^ |
| **CD205 expression levels by BAL CD11c+ cells (Mean fluorescence intensity)** | 12,784 ± 535 | 2,688 ± 137 *** |
| **CD205 expression levels by lung CD11c+ cells (Mean fluorescence intensity)** | 11,205 ± 436 | 3,100 ± 82 *** |

BAL cells and cell suspensions from left lungs were prepared from naïve wild type and IL-19-/- mice of the C57BL/6 strain. Cells were analyzed by flow cytometry. Data represent means ± Standard Error of Means of 15-21 mice per group. The data were pooled from 2 independent experiments. Statistical significance was calculated with the Mann-Whitney U test: ^ns^ not significant; *** p<0.0001.
